# To the Point

**Published:** 1871-05

**Authors:** 


### TO THE POINT.

A paper published in the present number of the REGISTER, entitled "Causes Retarding Dental Progress," suggests the immense difficulty some writers experience in getting to the point, or rather, in taking their readers to it. The writer roams over a vast field of intelligence, picking up here and there a fact, and holding it a moment for illustration; but, after traversing the entire area, fails to show why any single fact or the whole array of facts, retard dental progress.

If the head were changed to any other, the body of the article would fit quite as well. It is like the play of Hamlet with Hamlet left out. Indeed, it reminds us of the delighted father that named the baby before it was born, but after the event found that the name did not suit the sex.

The essayist, is however, not alone in this intellectual floundering; many excellent minds keep him company, and it is only because he is egregiously at fault in this respect, that his morsel is held upon the editorial fork for inspection. What would be thought of an operator whose plain and palpable duty it was to extract a tooth, yet, who instead of applying the forceps at once, should flourish around the patient with Dervish-like genuflections, and pinch his nose now and again with the instrument, touch the sensitive tooth a few times, just enough to drive the patient mad with pain, and finally dismiss him without performing the operation? This illustration may overstate the case of our essayist, but it is clear that there is too much sound in his index, and that he lays down his pen without taking up his subject.

To get at the point by the most direct route is the great need, and the true art of writing. All labored efforts are wearisome and unprofitable. To have something to say, and then to say it in the fewest forcible words is enough. It will give the most satisfaction in the end, and draw writer and reader into closer intimacy, and more enduring friendship. T.

